# Risk-Sensitive Optimal Feedback Control Accounts for Sensorimotor Behavior under Uncertainty

**DOI:** 10.1371/journal.pcbi.1000857

**Published:** 2010-07-15

**Authors:** Arne J. Nagengast, Daniel A. Braun, Daniel M. Wolpert

**Affiliations:** 1 Computational and Biological Learning Lab, Department of Engineering, University of Cambridge, Cambridge, United Kingdom; 2 Department of Experimental Psychology, University of Cambridge, Cambridge, United Kingdom; University College London, United Kingdom

## Abstract

Many aspects of human motor behavior can be understood using optimality principles such as optimal feedback control. However, these proposed optimal control models are risk-neutral; that is, they are indifferent to the variability of the movement cost. Here, we propose the use of a risk-sensitive optimal controller that incorporates movement cost variance either as an added cost (risk-averse controller) or as an added value (risk-seeking controller) to model human motor behavior in the face of uncertainty. We use a sensorimotor task to test the hypothesis that subjects are risk-sensitive. Subjects controlled a virtual ball undergoing Brownian motion towards a target. Subjects were required to minimize an explicit cost, in points, that was a combination of the final positional error of the ball and the integrated control cost. By testing subjects on different levels of Brownian motion noise and relative weighting of the position and control cost, we could distinguish between risk-sensitive and risk-neutral control. We show that subjects change their movement strategy pessimistically in the face of increased uncertainty in accord with the predictions of a risk-averse optimal controller. Our results suggest that risk-sensitivity is a fundamental attribute that needs to be incorporated into optimal feedback control models.

## Author Summary

In economic decision-making it is well-known that when decision-makers have several options, each associated with uncertain outcomes, their decision is not purely determined by the average payoff, but also takes into account the risk (that is, variability of the payoff) associated with each option. Some actions have a highly variable payoff, such as betting money on a horse, whereas others are much less variable, such as the return from a savings account. Whether an individual favors one action over the other depends on their risk-attitude. In contrast to economic decision-making, models of human motor control have exclusively focussed on models that maximize average rewards (minimize average cost). Here, we consider a computational model (an optimal feedback controller) that takes the variance of the cost into account when calculating the best movement strategy. We compare the model with the performance of human subjects in a sensorimotor task and find that the subjects' behavior is consistent with the predictions of a risk-sensitive optimal feedback controller with most subjects being risk-averse.

## Introduction

Risk-attitudes are an important determinant of human decision-making that expresses itself, for example, with individuals who are risk-seeking investing in highly volatile stocks and those who are risk-averse choosing governments bonds. Economic theory suggests that when making a decision that can lead to a probabilistic set of outcomes, each of which is associated with a different reward, decisions are not selected simply to maximize the average reward (expected value maximization). Instead both the average reward and the variability of the reward influence the decision. For example, when subjects are given a choice of either a risky but high-average reward (a 50-50 chance of winning 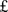
100 or nothing) and a sure-bet with lower average reward (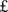
45 for sure), the majority of people choose the sure option – for example see [1], [2]. This effect is called risk-aversion because people are willing to accept a lower average payoff in order to reduce the variability of the payoff.

However, the motor system, unlike an economic decision-maker, has to act continuously in time and needs to incorporate incoming sensory information into the ongoing control process, e.g. in obstacle avoidance tasks [3]. Recently, optimal feedback control has been proposed as a model for continuous optimal decision-making and has successfully explained a wide range of movement phenomena such as variability patterns [4], the response of bimanual movements to perturbations [5], [6], adaptation to novel tasks [7]–[9] and complex object manipulation [10]. This model computes the optimal strategy given a cost function that penalizes a combination of error and effort. Although these motor control models take the stochastic nature of the task (arising from motor and sensory noise) into account, the potential effects of risk-sensitivity have been neglected. Specifically, optimal control models are risk-neutral in that they minimize the average cost. Here, we consider an optimal control framework that incorporates a risk-sensitive controller and use it to model subjects' behavior in a continuous decision-making task. We show that subjects' behavior is consistent with risk-sensitive optimal control models with most subjects being risk-averse.

## Results

**Figure 1:**
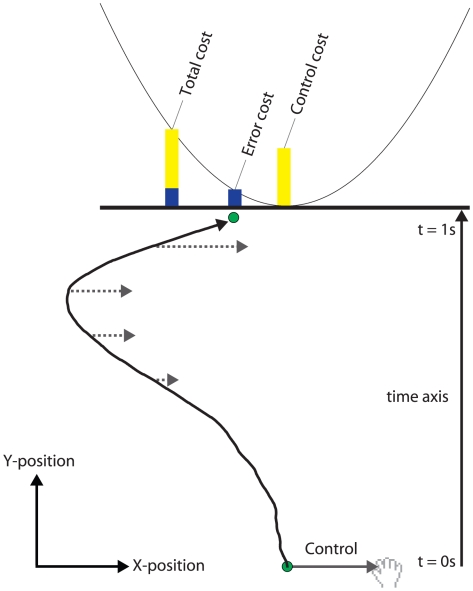
Schematic of the task. Subjects attempted to move a virtual ball (represented by the green circle) to the center of a target line (represented by the black horizontal line). The ball moved with constant y-velocity and hit the target after 1 s, whereas it moved with Brownian motion in the x-direction. Final positional errors were penalized by a quadratic cost function that was displayed as a parabola and the error cost was displayed at the end of the trial (blue bar). Subjects could exert control on the x position of the ball by moving their hand to the left or right (gray solid and dashed arrow lines). This incurred a control cost which was the quadratic in the control signal and the cumulative across a trial (yellow bar) was constantly displayed. At the end of the trial subjects received feedback of the total cost, the sum of control and error cost (yellow-blue bar). Subjects were required to minimize the total cost on average and were tested on four conditions (2 noise levels×2 control cost levels). The path taken by the ball is shown for a typical trial.

To examine risk-senstivity in sensorimotor control, we simulated the motion of a ball that moved with constant speed towards a target line (Figure 1). When the ball crossed the line, its deviation from the center of the line led to a quadratic penalty (error cost in points). The motion of the ball in the orthogonal direction was determined by two processes. First, random forces acted on the ball (drawn from a zero-mean Gaussian distribution) which caused the ball to drift under Brownian motion. Second, subjects could exert control on the ball by moving their hand left and right, with this deviation mapped linearly to a simulated force acting on the ball. Subjects were penalised quadratically for applying control to the ball and this control cost was cumulative over the movement. Subjects were instructed to minimize the total cost which was the sum of the error and control costs. Therefore, to minimize the total cost subjects wanted to come close to the center of the target line while exerting minimal control. We varied both the variance of the noise added to the force acting on the ball as well as the relative weighting of the error and control cost, leading to four conditions (1. low noise/low control cost, 2. high noise/low control cost, 3. low noise/high control cost, 4. high noise/high control cost).

**Figure 2:**
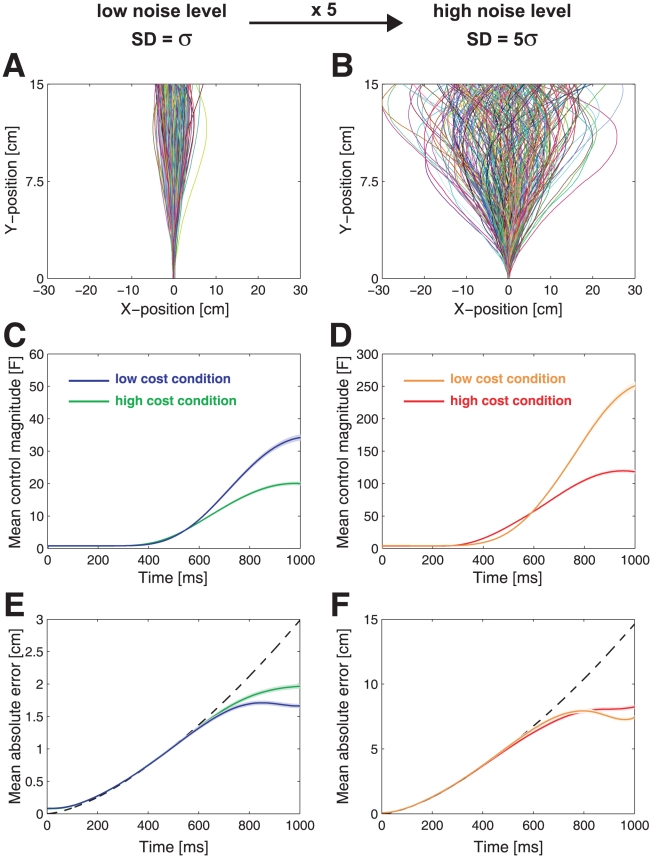
Task performance. A. All 250 ball paths for a typical subject for the low noise level (high control cost condition). Individual trials are colored randomly. B. as A. but for the high noise level. C. Mean control magnitude across all trials and subjects for the low noise level (blue - low cost condition, green - high cost condition). D. as C. but for the high noise level (yellow - low cost condition, red - high cost condition). E. Mean absolute positional error (absolute deviation from the center of the target line) across all trials and subjects for the low noise level (colors as in C.) The dashed line shows the mean absolute error if subjects did not intervene. F. as E. but for the high noise level. Note that the y-scale in D. and F. is five times greater than in C. and E. due to the higher noise level. Shaded area shows one s.e.m. across all trials.

Figure 2A & B depicts the ball's path for the two noise levels in the high control cost conditions for a typical subject. These show that the ball deviates initially due to the noise acting on it and clear corrections can be seen towards the end of the movement. Increasing the noise level (Figure 2B) led to the ball showing a wider distribution. Figure 2C & D depicts the mean control magnitude averaged across all subjects. This shows that subjects tended to apply increasing levels of control towards the end of the movement. Compared to the low noise condition, in the high noise condition subjects applied more control (repeated-measures ANOVA of the absolute control command at the end of the trial: 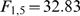, 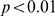). Similarly, when the cost of applying control was reduced (low cost condition), subjects applied more control to the ball in order to reduce the positional error (repeated-measures ANOVA: 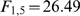, 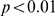). Figure 2E & F shows the mean absolute error over time averaged across all subjects. Simulations demonstrate that without any intervention (dashed lines) the final positional error for the high noise levels is, as expected, 5 times higher than for the low noise condition. Subjects applied control thereby reducing the positional error. The positional error at the end of the trial was smaller in the low control cost condition compared to the high control cost condition (repeated-measures ANOVA: 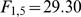, 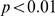). Taken together, the results suggest that subjects were flexibly adapting their strategy in the four different conditions and that they were sensitive to the relative weighting of the error and control cost settings.

**Figure 3:**
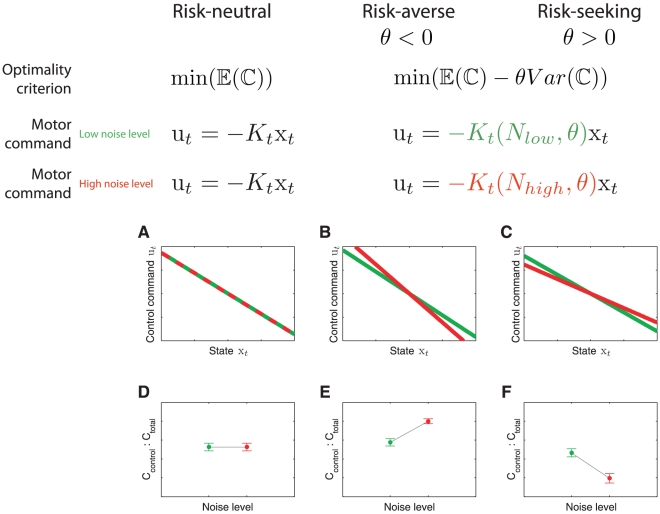
Predictions of optimal feedback control models. A risk-neutral optimal control model [4], [17] attempts to minimize the mean of the cost function. As a result, its policy (that is the motor command applied for a given state of the world) is independent of the noise variance N. In contrast, a risk-sensitive optimal control model [22], [34] minimizes a weighted combination of the mean and variance of the cost. Additional variance is an added cost for a risk-averse controller (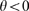), whereas it makes a movement strategy more desirable for a risk-seeking controller (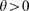). As a consequence, the policy of the controller changes with the noise level N depending on its risk-attitude 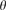. A.–C. Changes in motor command with the state of the ball (its positional deviation 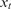 from the center) for a low noise level (green) and for a high noise level (red) for the risk-neutral (A), risk-averse (B) and risk-seeking (C) controllers. The slope of the lines is equivalent to the control gain of the controller. D.–F. Contribution of control cost to total cost (control cost+error cost) for the risk-neutral (D), risk-averse (E) and risk-seeking (F) controllers.

To examine whether subjects were risk-sensitive, we investigated the predictions of both a risk-neutral and a risk-sensitive optimal control model. Due to its altered cost function, which considers the mean and the variance of the cost (Figure 3), the risk-sensitive optimal control framework makes distinct predictions for the two levels of noise. In our case, a risk-neutral optimal feedback control law does not depend on the variance of additive noise (see Methods). Therefore, if subjects were risk-neutral, their control signal for a particular state of the ball should be independent of the noise level (Figure 3A). In contrast, a risk-sensitive optimal feedback control law depends explicitly on the variance of additive noise. In a risk-averse controller, for a given state, larger control signals should be applied with larger noise variance (Figure 3B). In contrast, in risk-seeking control the opposite pattern should be observed (Figure 3C).

**Figure 4:**
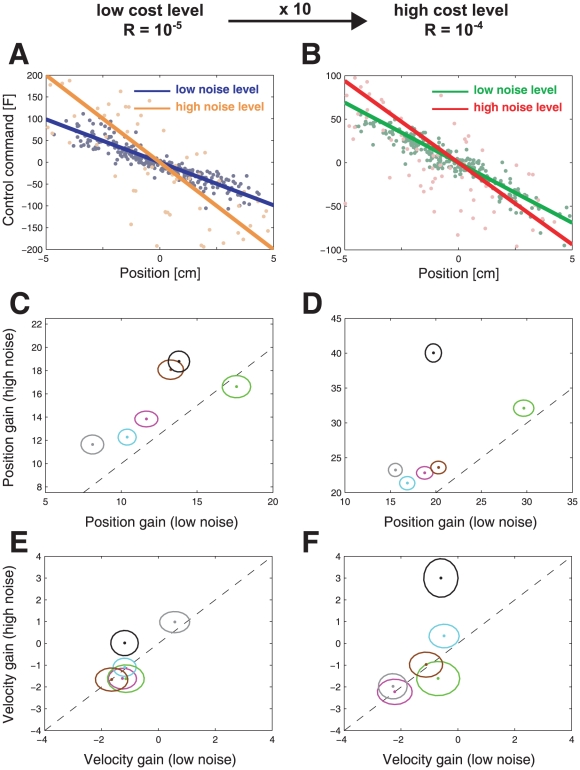
Risk-sensitivity. A. Results of the multilinear regression analysis of the low control cost conditions for subject number 5. The line shows the average motor command that the subject produces for a given position (blue - low noise level, yellow - high noise level). The slope of the line is a measure for the position gain of the subject. B. same as in A. but for the high control cost conditions (green - low noise level, red - high noise level). C.–F. Compares various measures between the high and low noise conditions. A risk-neutral controller predicts values to be the same for both condition (dashed line), a risk-averse controller predicts values to fall above the dashed line and a risk-seeking controller below it. C. Negative position gain for the high noise condition plotted against the low noise condition for all six subjects in the low control cost conditions (subject 5 in black, ellipses show the standard deviation). The dashed line represent equality between the gains. D. as C. but for the high control cost conditions. E. Negative velocity gain for the high noise condition plotted against the low noise condition for all six subjects for the low control cost conditions (ellipses show the standard deviation). F. as E. but for the high control cost conditions.

We used multiple linear regression to estimate how the control signal at one point in time (0.9 s into the 1 s ball motion) depended on the state of the ball (150 ms earlier – see Methods for details). For each subject and condition we fit the control signal as a function of the x-position and velocity of the ball. Figure 4A & B shows slices through the two-dimensional fit to the data for a typical subject. These linear fits had an 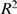 ranging from 0.62 to 0.88 (mean ± SD = 0.81±0.058; see Table 1 for values of each subject and condition). This allowed us to estimate the position and velocity control gains and test whether the control rule changed between conditions. A repeated-measures ANOVA on the positional gain of the control signal (factors of noise level and control cost) showed that there was a significant main effect of both noise level (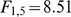, 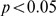) and of the cost (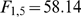, 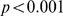) but no interaction. A similar analysis of the control signal's dependence on the ball's velocity showed no significant effects. These result are inconsistent with a risk-neutral controller in which the gains should be independent of the noise level. An analysis of the gain showed that it increased with the increased noise level as predicted by the simulation of the risk-averse controller (Figure 3B), and decreased with increased control cost showing that subjects' control laws were sensitive to the relative costs of control and final error.

**Table 1:** R^2^-values of the multiple linear regression analysis.

Subject	low control cost	low control cost	high control cost	high control cost
	low noise	high noise	low noise	high noise
1	0.79	0.82	0.88	0.87
2	0.88	0.83	0.85	0.82
3	0.79	0.82	0.74	0.82
4	0.82	0.87	0.85	0.87
5	0.89	0.85	0.83	0.82
6	0.64	0.85	0.73	0.84

R^2^-values of the multiple linear regression analysis on how the control signal at one point in time depended on the state of the ball (150 ms earlier) for each subject and condition.

**Figure 5:**
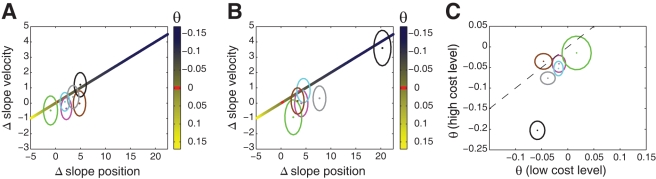
Difference in position and velocity gains. A. The difference in velocity gains plotted against the difference in position gains of all subjects for the low control cost conditions (ellipses show the 95% confidence region). The color gradient indicates the values predicted by the simulations of a risk-sensitive optimal controller for different 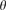-values. B. as A. but for the high control cost conditions. C. Subjects' individual risk-parameters 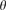 inferred from the experimental data of the high cost level versus 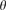 inferred from the data of the low cost level (ellipses show 1 s.d.).

In economic decision-making subjects tend to have their own individual risk-attitude. We therefore examined each subject's behavior to assess their risk-sensitivity. Figure 4C & D shows the position gain for each subject for the low and high noise condition and shows that five subjects had gains that were significantly increased in the high noise condition (F-test: all 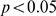) suggesting that they are risk-averse. For the high control cost conditions (Figure 4D) four of the subjects were still significantly risk-averse. A similar analysis of the velocity gain did not reach significance for any of the subjects (Figure 4E & F). However, the changes in velocity gain that we expect for a risk-sensitive controller are relatively modest compared to the change in position gain. Figure 5A & B shows the changes in velocity against position gain for simulations of an optimal controller with a range of risk-sensitivities. The data for the subjects fall approximately on the line predicted by the simulations of the risk-sensitive controller. All subjects except one fall in the range of a risk-averse controller. Thus failure to detect a significant change in the velocity gain from the low to the high noise level is not inconsistent with a risk-sensitive optimal controller. From the analysis depicted in Figure 5 we could also infer subjects' individual risk-parameters. Since we had a low cost and a high cost condition in both of which we manipulated the variability of the trajectories, we could use the two different cost conditions to infer subjects' individual risk-parameters from both conditions independently. This allows a consistency check as to whether subjects' inferred risk-parameters are similar in the two conditions. When we performed this analysis we found that the inferred risk-parameters were consistent for 5 out of 6 subjects (Figure 5c).

**Figure 6:**
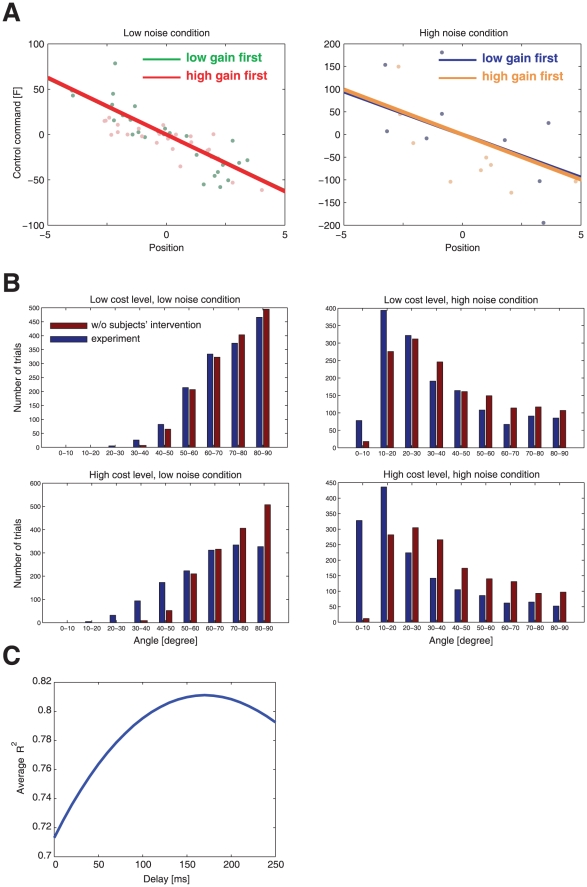
Analysis of possible confounds and sensorimotor delay. A. Results of the multilinear regression analysis of the first 15 trials for the low and the high noise condition. The subjects' data was pooled according to whether they began the experiment with a low gain or with a high gain (green and blue - low gain first; red and yellow - high gain first). B. Number of trials for different angles of the velocity vector of the ball with the wall upon impact (dark red - hypothetical impact angle of the ball had the subjects not intervened, dark blue - actual impact angle during the experiment). C. R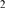-values of the multilinear regression analysis averaged across all subjects and conditions for different sensorimotor delays.

The observed change in control gains raises the question as to whether there are other possible explanations that do not depend on risk-sensitivity. We considered three possible alternatives. First, we investigated whether the different gains in the high- and low-noise condition could explain our results. In our experiments, hand positions were translated linearly into a virtual force acting on the ball. The gain of this linear relationship was decreased in the high-noise condition, so that the range of hand movements was similar in both noise conditions in order to avoid effects of different physical effort and signal-dependent noise (see Methods). However, incomplete adaptation to the different gains might have also led to over- or under-compensated movements. Importantly, it should be noted that the gains are not visuomotor gains between hand- and cursor-positions, that is a gain of 1 does not have any special meaning in our case. Even if subjects adapted incompletely this would not affect our analysis, as we are only interested in the change of slope between two different noise conditions. For an incomplete adaptation the slope would be different compared to complete adaptation, but as long as there is similar levels of adaptation to all gains this does not affect the conclusions. Yet, if we assume that there are different degrees of incomplete adaptation to the different gains, then this could lead to over- or under-compensated movements. Although it is not clear why such a strongly non-linear relationship should hold between different gains and the respective adaptations (since we deal with arbitrary position-to-force mappings), we tested for this possible confounding effect in our data during the initial training phase. This was possible because the order of the high- and low-noise (and therefore low- and high-gain) was randomized across subjects. Specifically, for the high cost level the randomization resulted in 3 subjects first experiencing the low gain condition and 3 subjects first experiencing the high gain condition. For the low cost condition the randomization resulted in 6 subjects first experiencing the low gain condition and 0 subjects first experiencing the high gain condition. Due to its even distribution we therefore only analyzed the high cost condition in the following. If our results were to be attributed to incomplete adaptation to different gains, we would expect to see that subjects that underwent a transition from high to low gains should show adaptation effects different to subjects that underwent a transition from low to high gains. In particular, we would expect that somebody who transits from high gains to low gains should have a tendency to under-compensate (reduction in slope in our experiment), whereas somebody who transits from low gain to high gains should have a tendency to over-compensate (increase in slope). However, when we compared the first 15 trials of the high-gain condition for subjects that started with this block to the first 15 trials of the high-gain condition for subjects that had already experienced the low-gain block, we found no statistical difference or bias. Similarly, we found no difference for the low gain condition for the two groups that experienced it in a different order (Figure 6a). Thus, we conclude that the differences in control gains that we observed for the different noise conditions in Figure 4 do not arise form the different gains.

Second, we examined whether our results could be explained by a different performance criterion. It is known in grasping, for example, that subjects prefer to have orthogonal angles of approach of each finger to an object [11], [12]. The biomechanical reason is that the object is most stably grasped when the finger approach orthogonally (as for example noise has minimum effect then and the force acts orthogonally and therefore does not promote slip). In our experiment it is not clear why an orthogonal approach would be beneficial. However, we might speculate that subjects might have tried similarly to achieve orthogonal impact with the ball at the wall. Since in the high-noise condition the number of orthogonal impacts is reduced due to the higher variance in velocity, subjects might have incurred more control costs to achieve the same level of orthogonal impacts. In this alternative explanation the assumption is that subjects control the ball in a way that makes it more likely to hit the wall orthogonally. To test this hypothesis we examined how often the ball hit the wall orthogonally (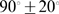) in the experiment and compared it to how often the ball would have hit the wall orthogonally if subjects had not intervened. This latter quantity could be computed from the experimental data as well, since the noise that drove the ball in the experiment was added independently at each time point, thus giving us access to the state, the control signal and the noise at each time point. Contrary to the orthogonality explanation, we found that subjects decreased the probability of an orthogonal impact on the wall by their interventions in all four conditions (repeated measures Anova with/without control and noise level as factors: significant main effect for with/without control (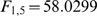, 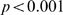) and of the noise level (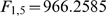, 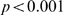), see Figure 6b). This suggests that an orthogonal impact angle was not an important determinant of subjects' behavior, since based on this criterion they could have performed better by not doing anything at all. When we conducted a similar analysis but only considered trials in which the ball hits close to the center of the wall (±2.5 cm), the conclusions remained the same.

**Figure 7:**
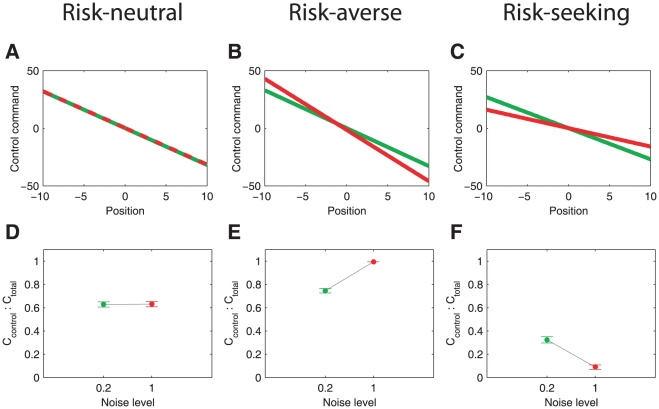
Simulations of an optimal controller with incomplete state observation and sensorimotor delay. A.–C. Changes in motor command with position for a fixed velocity (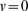) for the low noise level (green) and for the high noise level (red). D.–F. Contribution of control cost to total cost (control cost+error cost). A. & D. - Predictions of a risk-neutral controller. B. & E. - Predictions of a risk-averse controller. C. & F. - Predictions of a risk-preferring controller.

As a third explanation we considered the influence of observation noise. So far we have only considered the predictions of a risk-neutral and a risk-sensitive controller with no sensorimotor delay and complete state observation, that is assuming perfect knowledge of the position and the velocity of the ball. However, when investigating which sensorimotor delay can explain the relationship between state and control the best, we found that a delay between 150 ms and 200 ms lead to the highest R^2^ values (Figure 6c). To control for the possibility that a risk-neutral controller with observation noise and sensorimotor delay could explain the experimental data, we ran optimal control simulations with the parameters used in the experiment. Figure 7 shows the predictions of an optimal controller with incomplete state observation with a physiological valid sensory noise level [4] and sensorimotor delay [13]–[15]. Although optimal estimation is not independent of the process noise level (see Methods) even in the risk-neutral case, the predictions of this extended model do not differ appreciably from the case of complete observation. We also included observation noise on the target position (either with the same magnitude as the observation noise on the position of the ball or ten times larger), which did not lead to any significant differences in the control gains of a risk-neutral controller (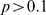 *,* 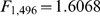 *).*

**Figure 8:**
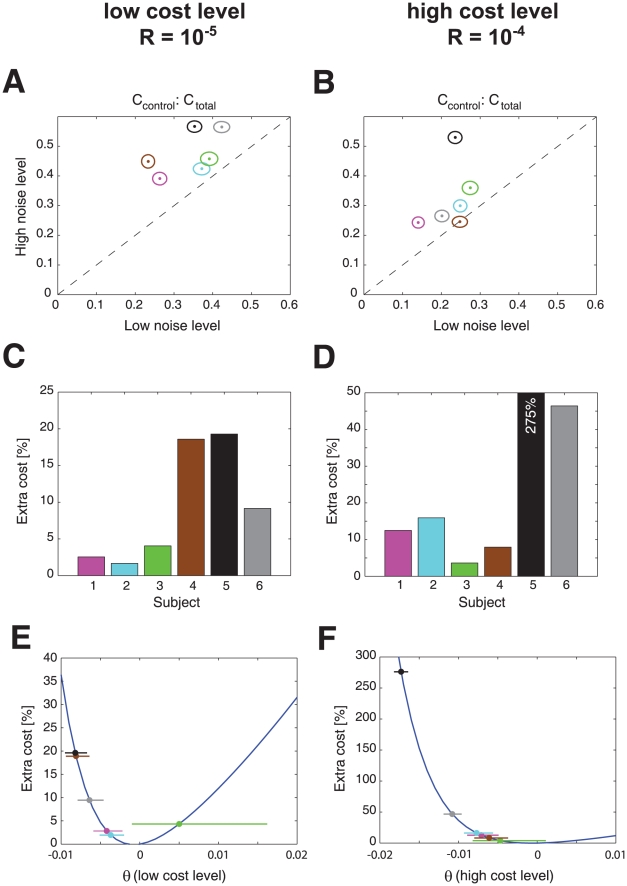
Contribution of control cost to total cost, and extra cost from risk sensitivity. A. Contribution of control cost to total cost for the high noise condition plotted against the low noise condition for the low control cost conditions (ellipses show 1 s.e.m. across all 250 trials). B. as A. but for the high cost level. C. Estimated extra cost in percent of a risk-sensitive controller with incomplete observation and sensorimotor delay based on the experimentally inferred 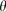-parameters for the low cost level. D. as C. but for the high cost level. E. Relationship between extra cost of a risk-sensitive controller relative to a risk-neutral controller for a range of 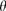-values overlaid with the subjects' experimentally inferred 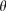-parameters 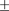 standard deviation for the low cost level. F. as E. but for the high cost level.

A final feature of risk-sensitive control that we examined is the noise-dependence of the trade-off between the control and error cost. Simulations show that a risk-neutral controller will have a total cost that is on average made up of a fixed proportion of control and error cost and this proportion is independent of the noise level (Figure 3D). In contrast, the relative contribution of control cost to the total cost increases with the level of noise for a risk-averse controller (Figure 3E). Conversely for a risk-seeking controller the relative contribution of control cost to the total cost decreases with the level of noise (Figure 3F). A repeated measures ANOVA on the control cost contribution (factors of noise level and control cost) showed that there was a significant main effect of both noise level (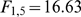, 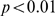) and of the cost (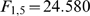, 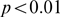) but no interaction. The control cost contributed relatively more to the total cost when the variance was increased or the control cost was reduced. Again this is consistent with a risk-averse control policy. Figure 8A & B shows the proportion of total cost that arises from the control cost for a low and high variance condition for the two cost levels. This shows that five subjects increased their control cost contribution (two-sample t-test: all 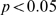) when the variance increased in the low cost condition and four increased the control cost contribution in the high control cost condition. This effect is a direct consequence of a risk-averse cost function which considers movement strategies with higher cost variance as less favorable. Movement cost is a combination of error cost (which is highly variable due to noise) and control cost (which is certain as the subject can set it to any level it likes). Hence, participants expended relatively more cost on control than on error in the high noise condition reducing the variable error cost at the expense of a certain control cost. Since all risk-sensitive subjects showed risk-averse behavior, our results suggest that even though the ball was in the same state, the risk-averse subjects tried to move it more strongly towards the center of the target line when the noise level was higher. Subjects were even prepared to accept lower payoffs in order to avoid highly uncertain trajectories. To quantify this extra-cost accepted by subjects we simulated a risk-sensitive optimal feedback controller with incomplete state-observation and a sensorimotor delay of 150 ms whose risk-sensitivity was tuned to the subjects' inferred individual risk-parameter. We then compared the total costs that were incurred by the risk-sensitive control scheme to a risk-neutral optimal controller. The percentage extra-cost accepted by subjects with the experimentally inferred risk-sensitivity can be seen in Figure 8C–F. As the ball's motion was perturbed by Gaussian noise it is as likely to drift towards the center as it is to drift away and this is independent of the noise variance. Hence, the subjects acted as if the noise would turn out to their disadvantage and their behavior reveals a pessimistic attitude towards uncertainty.

## Discussion

In our study we found that subjects' movement policy is sensitive to the variance of the cost and that the observed changes in behavior can be explained by the predictions of a risk-sensitive optimal controller. We tested subjects in a sensorimotor experiment in which they had to control a virtual ball undergoing Brownian motion. Subjects were required to minimize an overall cost that was the sum of a final positional error cost and an integrated control cost. We tested subjects in two conditions that differed in the variance of the noise acting on the ball. In a first analysis, we examined whether subjects changed their movement strategy between these two conditions and found that most subjects applied larger control commands for the same state of the ball in the higher variance condition. An analysis on a subject-by-subject basis showed that subjects' behavior was in accordance with the predictions of a risk-averse controller. In a second analysis, we examined how subjects trade off error cost against control cost in the two variance conditions. We found that subjects accrued relatively more control cost in the higher variance condition, that is they reduced error cost at the expense of control cost. Again, these findings are in line with the predictions of a risk-averse controller. Together with our previous results [16] this suggests that in sensorimotor control subjects take the variability of cost into account. Additionally, our current study shows the importance of risk-sensitivity in time-continuous tasks that are typical for motor control, and it provides evidence that risk-sensitive optimal feedback control is necessary to understand behavioral changes in response to changes in uncertainty. These findings are inconsistent with a risk-neutral account of motor control.

Previous studies have used risk-neutral optimal control models to explain a wide-range of movement phenomena [17], [18]. According to these optimal control models, an optimal movement plan optimizes a performance criterion given a set of task goals, and the noise properties and dynamics of the system under control. The performance criterion is typically chosen as the expectation of a quadratic cost function. Crucially, the optimal movement strategy (such as a feedback rule) suggested by such models is independent of the variance of the cost and exclusively considers its expectation. The omission of cost variance should, however, not be confused with the variance of the movement outcome (i.e. variability of trajectories [4]) that a risk-neutral optimal controller does take into account – for example, see [19], [20]. To formalise the difference between the variability in outcome and the variance of the cost we can consider the basic mathematical structure of optimal feedback control models. Consider a sensorimotor system with state 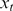 acted upon by a control command 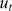. An effort-accuracy trade-off given by the final movement error (weighted by 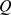) and the magnitude of the effort (weighted by 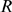), can be written as a quadratic cost function:
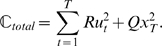
(1)However, as the system is stochastic due to noise in the control loop, the cost will also be variable for any given control law and therefore an optimal controller can only sensibly optimize some statistical property of the cost (such as the mean):
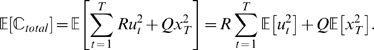
(2)This expectation value of the quadratic cost takes into account the variance of the movement trajectories and the control signal [20], [21], since 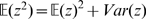. In our case Eq. 2 even reduces to a minimum-variance model [19] of positional error and of control, as the expectation of both final position and control command is zero:
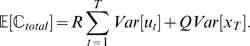
(3)In contrast, a risk-sensitive optimal controller minimizes the following cost function
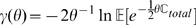
(4)which considers the variance of the cost and other higher-order moments of the cost [22], [23], which can be seen if we take its Taylor Series expansion
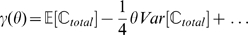
(5)Thus, this risk-sensitive model considers the expectation value of the cost and the entailed variance of movement trajectories just like the risk-neutral controller. Additionally it considers higher-order moments of the cost which are not taken into account by a risk-neutral controller. The risk-sensitive model (Eq. 4) provided a good explanation for subjects' behavior in our experiments, whereas a risk-neutral account (Eq. 2) failed to do so in most cases.

Although the risk-sensitive controller minimizes a different cost function, it still assumes quadratic payoffs measured by 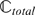. This raises the question in how far risk-sensitivity reported in our study depends on this particular form of the experimentally imposed quadratic cost function. It should be noted that risk-sensitivity always depends on the coordinate system chosen. People are risk-sensitive with respect to money (Bernouilli's famous log-utility curve [24]), for example, but of course they are not risk-sensitive then with respect to the logarithm of money. In our model we assume a particular cost function that subjects have to internalize. While this does not give a quantification of risk-sensitivity without any assumptions, our experiment clearly shows that a risk-neutral optimal feedback controller with quadratic costs (the standard model in the literature) cannot explain our data. However, we can explain our data with a risk-sensitive optimal feedback controller that assumes quadratic costs. This raises the question whether there are other cost functions that could account for our data, for example the robust cost function seen in pointing behavior that is quadratic locally but then levels off [25]. Such a robust cost function cannot explain the risk-averse behavior observed in our subjects as, rather than being sensitive to large errors, it discounts them thereby encouraging risk-seeking behavior. Another important assumption is that the subjective cost function stays the same over the various conditions. This assumption does not seem to be unreasonable, as the task stayed the same and only the statistics changed. We also found in previous experiments [10] that we could describe motor behavior with a wide range of different dynamics with the same cost function, as long as the task stayed the same.

Previous studies have shown that risk-sensitivity in an individual is highly context-dependent and can change across situations (see, for example, [26]–[29]). Thus, if we were to assess our subjects' risk-sensitivity in another task, the outcome could differ. However, we conducted a consistency check within our experiment, as we had two cost conditions (high and low) in which we manipulated the variance. This showed that the two deduced risk-sensitivities were largely consistent, at least within our task. There are a few differences to previous optimal control studies that are worth noting. First, one key difference of previous optimal control studies compared to our study is that the noise level was not altered systematically, making it difficult to establish the influence of variance on subjects' behavior. Second, by changing the gain between the hand position and control command between the high and low noise conditions, our experiment minimized the effects of multiplicative noise. In free movements signal-dependent noise is an important determinant of people's movement strategy and a risk-neutral optimal control model predicts policy changes if the variance of this signal-dependent noise is altered [4]. Currently, there exists no closed-from solution for an optimal controller with signal-dependent noise for the risk-sensitive case and it will be a future challenge to devise risk-sensitive control models that can deal with multiplicative noise. Third, previous studies assume an implicit cost function which is quadratic in accuracy and effort terms, and the relative contribution of the two terms is either fit to the data or set a priori to some reasonable level. As we wished to avoid fitting the cost parameters, we imposed an explicit cost function on the task that allowed us to manipulate the relative weighting between control cost and state cost, and see how much cost subjects accrued in different noise conditions. Although some studies have attempted to estimate subjects' cost functions [20], [25], a promising approach will be to refine algorithms (inverse optimal control models) that could directly infer people's cost functions from human movement data. Fourth, a type of uncertainty that we have not studied is the noise in the sensors that ultimately limits the accuracy of our vision and proprioception. Our study was designed so that the magnitude of the observation noise was negligible in comparison to the process noise level reference. In the future, it might be interesting to study risk-sensitivity under observation noise and to investigate how estimation and control processes interact in a risk-sensitive manner [22], [30].

One of the cornerstones of optimal control theory is its flexibility to consider several objectives in the movement strategy which correspond to a trade-off between the different terms in the cost function. As we used explicit (points) rather than implicit cost (error and effort), we were able to directly test whether subjects were sensitive to manipulations of the importance of one of the cost terms. We found that subjects exerted more control and reduced their positional error in the conditions where the control cost was reduced. Furthermore, we also estimated the subjects' policy for a given position of the ball and could show that subjects increased their positional gain in the low control cost conditions. Hence, subjects adapted their strategy flexibly to the lower movement cost and increased their position gain to decrease their positional error which had become relatively more important in the cost function. Previous studies have demonstrated other aspects of task flexibility in human motor control using optimal control models. Recently, the goal dependence of bimanual movements was shown experimentally to be in line with the predictions of two controllers acting either towards the same or two distinct goals [5]. Similarly, a single controller can incorporate additional task goals such as a stability requirement [3] or object manipulation [10], and subjects adapted their movement strategy flexibly to the new task requirements as predicted by extended optimal control models.

Here, we demonstrate that the economic concept of risk can be applied to computational models of motor control and that it is necessary to understand human movement behavior in response to changes in uncertainty. Our results could also be interpreted as evidence for robust control, since there is a close theoretical relationship between risk-sensitive control and robust control. A robust controller is able to keep a control process stable within certain error bounds even if the assumed forward model is uncertain or wrong. Such a controller acts like a risk-averse controller [31], [32] by putting a lower bound on ‘how bad things could get’. Thus, the risk-parameter could also be interpreted as a robustness parameter. In the future, it will be interesting to investigate whether other robust control principles can be applied to human movement control.

## Methods

### Ethics Statement

All experimental procedures were approved by the local ethics committee and subjects provided written informed consent.

### Experimental Procedures

Six healthy right-handed participants (two female, four male, average age 25 yrs) took part in the study. Subjects held the handle of a vBOT robotic manipulandum that could be moved with minimal inertia in the horizontal plane [33]. The position of the vBOT handle (i.e. the hand) was calculated online at 1000 Hz. The arm was hidden from view and a mirror rear-projection system was used to display visual images in the plane of the arm.

The task required the subjects to steer a small circular ball (displayed as a 0.1 cm radius cursor) to a horizontal target line situated 15 cm from the initial ball location (Figure 1). On each trial the ball moved with constant y-velocity towards the target at 15 cm

s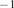 and the trial ended when the ball reached the target line after 1 s. The motion of the ball in the x-direction was simulated as a frictionless mass (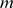 = 1 kg) and the force acting on the ball was determined by two additive processes. First, the ball acted under Brownian motion in which a random component of the force was drawn from a Gaussian zero-mean noise distribution 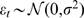. Second, subjects could exert control on the ball by moving their hand left and right. The position of the hand relative to its starting location was mapped linearly to a force acting on the ball. Therefore the equations of motion which were updated with a time step of 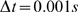 are given by
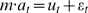
(6)

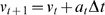
(7)

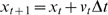
(8)where 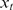, 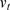 and 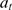 are the position, velocity and acceleration of the ball in the 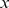-direction at time 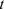, respectively. The visual display of the ball was updated at the screen refresh rate of 60 Hz. Subjects were required to perform the task so as to minimize an explicit cost that had two components. First, the control exerted by the subject incurred an instantaneous (cumulative) control cost that was quadratic in the control force (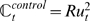). Second, there was an accuracy cost determined by where the ball crossed the target line relative to the midpoint of the target line. Subjects were penalised quadratically in this error 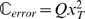. The total cost on a trial was therefore given by
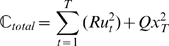
(9)


Both components of this cost were displayed graphically during each trial. The positional error function was displayed as a parabola whose height above the target line displayed the error that would accrue if the ball ended at that location on the line. Accordingly, the midpoint of the target line was marked by the minimum of the parabola. During the trial the cumulative control cost was also displayed and shown both numerically and graphically as a yellow bar in the same dimension as the positional error function (Figure 1). At the end of each trial the actual error cost incurred on that trial was shown numerically and graphically as a blue bar. Finally, control cost and error cost were added up graphically and numerically yielding the total cost. The numerical representation was referred to as points when subjects were instructed. Subjects were told to minimize their average number of (total) points and the average across all trials was displayed in the upper right corner.

Each subject performed the task with two different cost schemes (relative values of 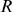 and 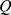) and under two different noise levels (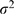) giving four conditions in total. In the high control cost condition 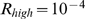 and in the low control cost condition 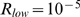 (for both conditions 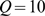). In the low noise condition 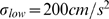 and in the high noise condition 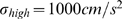. To normalize the distance that the hand was required to move to achieve the task, the gain of the linear mapping of hand position to control force was set to *k* = 10 N·cm^−1^ in the low noise condition and 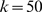 N

cm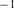 in the high noise condition. This controlled for the effects of signal-dependent noise and the intrinsic effort of moving the hand which were not included in the optimal control model (see below).

Half the subjects began with 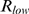, the other with 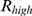. The order of 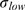 and 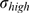 for a given control cost condition was randomized. First, subjects completed a training session of 50 trials at every noise level. Subsequently, the test session consisted of a block of 300 trials at every noise level. Hence, subjects completed a total of 1400 trials (4 conditions with 350 trials each).

### Data Analysis

The last 250 trials of each combination of settings was analyzed so as to exclude adaptation effects arising from the transition to different noise and cost environments. Our first analysis was designed to establish whether the control policy used by the subjects changed between conditions. Subjects tended to apply increasing control signals throughout a trial (Figure 2 C & D) and, therefore, we chose to analyse how the balls position and velocity late in the movement (t = 0.75 s) affected subsequent control. Due to intrinsic delays in the visuomotor system, which are of the order of 100–200 ms [13]–[15], we examined how the ball's state at 0.75 s into the movement affected the control signal generated 150 ms later. To quantify a subject's policy in a condition we regressed the control generated at t = 0.9 s as a function of the ball's x-position and velocity at t = 0.75 s. This multiple linear regression yields a plane in state-control space (the intercept is assumed to be zero and was not fit).

To establish whether the policy planes of two particular conditions differ, we compared nested models. The full model involved jointly fitting two conditions each with separate coefficients for position and velocity (4 predictor variables). Two reduced models were considered, one in which the two conditions shared the same dependence on position and one in which they shared the same dependence on velocity (both reduced models have 3 predictor variables). Model comparison was performed using an F-test on the sum of square errors of the two regression models.

### Optimal Control Models

We focus our analysis on using an optimal control model with complete state observation and no sensorimotor delays. Introduction of a sensorimotor delay and of physiological sensory noise into the simulations does not change the predictions of the models appreciably. The magnitude of the observation noise was negligible compared to the magnitude of the process noise level.

#### Risk-neutral optimal controller

A risk-neutral optimal feedback controller minimizes the expectation of the quadratic cost function 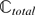 given a movement duration 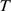, the constraints of the system dynamics and a movement goal. The state space of our system 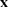 includes the x-position 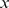 and the x-velocity 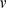 of the ball (the target position is at 0). The system dynamics in our case can be written in the form 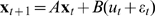 and in our case:
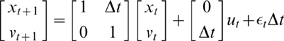
where 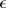 is normally distributed with a zero mean and covariance matrix
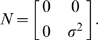
Consequently, the matrix form of the cost function is given by:
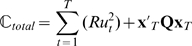
(10)where
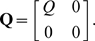
The feedback control law that minimizes the cost is:
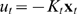
(11)where
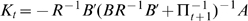
(12)and
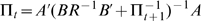
(13)where 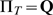. Note that the process noise variance 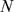 does not enter any of the equations that compute the optimal feedback law, which is why the policy of a risk-neutral controller is independent of the process noise level.

#### Risk-sensitive optimal controller

A risk-sensitive optimal feedback controller [22], [34] minimizes the following criterion function:
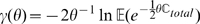
(14)where 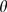 is a scalar that indicates the risk-sensitivity of the controller (risk-neutral as before for 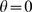, risk-averse for 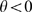, risk-seeking for 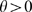). The first two terms of the Taylor Series expansion of 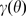 are 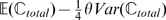 which corresponds to a simple risk-sensitive mean-variance decision-maker. The change in the cost function results in a modified form of the Ricatti recursion
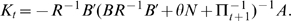
(15)and
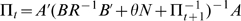
(16)The equations differ from the risk-neutral version by the addition of the risk-sensitivity parameter 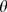 multiplied by the process noise level 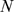. In general, the magnitude of 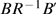 can be thought of as a measure of ‘control power’ that reflects a ratio between the control effectiveness as measured by 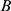 and the control cost as measured by 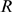 that is the control power is high for large 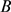 and small 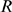 because it implies an increased and inexpensive influence of the control signal on the system. A risk-averse controller (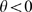) effectively reduces the overall ‘control power’ such that the controller acts as if the process noise directed the state in an undesired direction (pessimism). In contrast, a risk-seeking controller (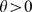) reflects an increase in ‘control power’ and the noise is perceived to bias the state in a desired direction (optimism). In the risk-neutral case (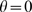) the equations reduces to the ones described in the previous section.

#### LQR with incomplete state observation and sensorimotor delay

So far we have only considered an optimal controller with perfect state estimation and without a sensorimotor delay. In the following, we describe the changes to the controllers that are necessary to include the two.

Risk-neutral case: We adapted the optimal control model described above in accordance with [4], [22], [34]. This was done by, first, changing the model from one of complete state observation to one of incomplete state observation:
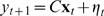
where 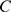 is the observation matrix and 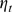 is a sensory noise term with mean 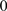 and covariance matrix 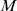. Second, a sensorimotor delay of a total of 15 time steps (i.e. 150 ms, which is roughly the time to respond to a visual perturbation [13]–[15]) was implemented using an augmented state [4], [10]. To obtain an optimal estimate of 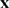 from only observing 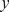, a Kalman filter combines a forward model prediction of 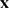 with the feedback information 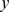. The state estimate is computed as
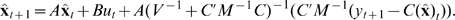
Note that, even in the risk-neutral case, the estimate covariance 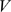 does depend on the level of process noise 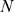:
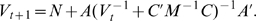
Since the control command 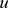 is a function of the estimate 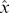 changing the process noise could potentially influence the controller. However if, as in our case, the magnitude of the observation noise is negligible compared to the process noise, this does not lead to an appreciable effect (see Simulations).

Risk-sensitive case: Due to the occurrence of the 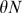 term in the equations for computing the risk-sensitive policy the separation between state estimation and optimal control is not complete. Nevertheless, a risk-sensitive certainty equivalence principles exists [30] and state estimation and optimal control can be coupled through
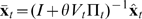
where the optimal control rule is now
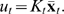



### Simulations

The same parameter settings were used in the simulations as in the actual experiment except where values had to be rescaled due to the different discretization which was used for computational reasons (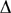
t = 1 ms in the actual experiment, 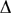
t = 10 ms in the simulations). Simulations were run 250 times with 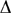
t = 10 ms and T = 1000 ms (i.e. 100 time steps). The rescaled process noise for 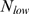 was 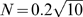 and for 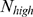 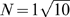. The error cost parameter was set to 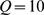 as in the experiment, and we only simulated the high control cost condition and rescaled the control cost parameter to 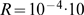. For the risk-neutral controller we set 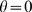, for the risk-averse we set 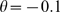 and for the risk-preferring we set 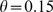. To obtain the control policy we used the same approach as for the experimental data. For the LQR with incomplete state observation and sensorimotor delay, the sensory noise terms were all set to 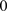 except for the ball's x-position and x-velocity which were set to 0.5 cm and 5 cm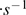 respectively [4].
